# Norepinephrine Induces PTSD-Like Memory Impairments *via* Regulation of the β-Adrenoceptor-cAMP/PKA and CaMK II/PKC Systems in the Basolateral Amygdala

**DOI:** 10.3389/fnbeh.2019.00043

**Published:** 2019-03-06

**Authors:** Xiang-Hui Liu, Rong-Ting Zhu, Bo Hao, Yan-Wei Shi, Xiao-Guang Wang, Li Xue, Hu Zhao

**Affiliations:** ^1^Faculty of Forensic Medicine, Zhongshan School of Medicine, Sun Yat-sen University, Guangzhou, China; ^2^Guangdong Province Key Laboratory of Brain Function and Disease, Zhongshan School of Medicine, Sun Yat-sen University, Guangzhou, China; ^3^Guangdong Province Translational Forensic Medicine Engineering Technology Research Center, Zhongshan School of Medicine, Sun Yat-sen University, Guangzhou, China

**Keywords:** PTSD, fear conditioning, basolateral amygdala, norepinephrine, AMPA, cAMP/PKA, CaMK II/PKC

## Abstract

Glucocorticoids (GCs) can modulate the memory enhancement process during stressful events, and this modulation requires arousal-induced norepinephrine (NE) activation in the basolateral amygdale (BLA). Our previous study found that an intrahippocampal infusion of propranolol dose-dependently induced post-traumatic stress disorder (PTSD)-like memory impairments. To explore the role of the noradrenergic system of the BLA in PTSD-like memory impairment, we injected various doses of NE into the BLA. We found that only a specific quantity of NE (0.3 μg) could induce PTSD-like memory impairments, accompanied by a reduction in phosphorylation of GluR1 at Ser845 and Ser831. Moreover, this phenomenon could be blocked by a protein kinase A (PKA) inhibitor or calcium/calmodulin-dependent protein kinase II (CaMK II) inhibitor. These findings demonstrate that NE could induce PTSD-like memory impairments *via* regulation of the β-adrenoceptor receptor (β-AR)-3′,5′-cyclic monophosphate (cAMP)/PKA and CaMK II/PKC signaling pathways.

## Introduction

Post-traumatic stress disorder (PTSD) is a serious anxiety disorder that usually follows a life-threatening traumatic event. The primary diagnostic criteria are re-experiencing of the trauma, autonomic reactivity to response, avoidance of trauma-related cues and elevated arousal (Long et al., 2013). A core symptom of PTSD is dysregulated fear response that is characterized by an over-generalization of fear and in tandem an inability to inhibit fear responses in the presence of safety (Jovanovic et al., 2012). Kaouane et al. (2012) developed an animal behavioral model that infusion of glucocorticoids (GCs) into the hippocampus in the predicting-context group conditioned with a high-intensity shock induced PTSD-like memory impairments, which were manifested as decreased freezing to the correct predictor and generalized fear responses to the cues that were normally not a relevant predictor of the threat. This animal model evaluates the ability of subjects to restrict fear responses to the appropriate predictor of a threatening stimulus, innovatively induced the core symptoms of PTSD; however, the neurobiological mechanisms underlying pathological PTSD-like memory impairment remain unclear.

GCs, steroid hormones from the adrenal cortex during stressful events, can modulate the memory enhancement process following an inverted-U shape dose-response relationship (Roozendaal, 2000; Sandi and Pinelo-Nava, 2007; Barsegyan et al., 2014; Deppermann et al., 2014; Finsterwald and Alberini, 2014). Additionally, this modulation requires arousal-induced norepinephrine (NE) activation in the basolateral amygdale (BLA; Roozendaal et al., 2004, 2006a,c), which is a key component of the neuronal circuits mediating emotional arousal and stress hormone effects on cognitive functions (McGaugh, 2004; Huff et al., 2006; Ehrlich et al., 2009; McIntyre et al., 2012). Evidence, including anatomical (Pikkarainen et al., 1999), behavioral (Roozendaal, 2000; Roozendaal et al., 2006c; McReynolds et al., 2010, 2014), electrophysiological (Akirav and Richter-Levin, 1999; Almaguer-Melian et al., 2003; Vouimba and Richter-Levin, 2013), and optogenetic data (Nabavi et al., 2014; Redondo et al., 2014; Tanaka et al., 2014; Rei et al., 2015), has shown that the amygdala modulates hippocampal memory storage. One of our recent studies found that an intrahippocampal infusion of propranolol dose-dependently induced PTSD-like memory impairments, demonstrating that the NE system in the hippocampus was involved in the formation of PTSD-like memory impairment (Zhu et al., 2018). However, whether NE within the BLA is a key factor in PTSD-like memory impairments remains unclear.

Numerous studies have demonstrated that NE activates adenosine 3′,5′-cyclic monophosphate (cAMP), cAMP-dependent protein kinase A (PKA; Roozendaal et al., 2002) and calcium/calmodulin-dependent protein kinase II (CaMK II) *via* β-adrenoceptor receptors (β-ARs) in the emotional memory (Hu et al., 2007). In contrast, other findings have shown that PKA activation, but not CaMK II activation, in the BLA is critical for cocaine memory restabilization processes (Arguello et al., 2014). Therefore, whether NE activates the β-AR-cAMP/PKA or CaMK II/PKC signaling pathway in PTSD-like memory impairments within the BLA remains to be elucidated.

Simultaneously, NE acting through β-ARs has powerful effects on the induction of long-term potentiation (LTP), which is one type of synaptic plasticity that has been linked to memory storage (Roozendaal et al., 2006b; O’Dell et al., 2010). Synaptic insertion of GluR1 subunit-containing α-amino-3-hydroxy-5-methyl-4-isoxazoleproprionic acid (AMPA)-type receptors (AMPARs) appears to have a critical role in the synaptic strengthening observed during LTP induction (Lee et al., 2003; Hu et al., 2007). AMPARs are ionotropic glutamate receptors generated by the combination of four subunit proteins known as GluR1, GluR2, GluR3, and GluR4 (Traynelis et al., 2010). Prior findings showed that LTP was reduced in GluR1 gene inactivation mice and phosphomutant mice with knock-in mutations of the GluR1 phosphorylation sites (Jensen et al., 2003; Hu et al., 2007). Consequently, phosphorylation of AMPA receptor GluR1 subunits has a key role in β-AR-mediated enhancement of both LTP and behavioral learning. Furthermore, GluR1 subunits have many phosphorylation sites in the intracellular C-terminal domain; the central role of phosphorylation of Ser831 and Ser845 sites has been well elucidated. Ser831 is phosphorylated by PKC as well as other kinases such as CaMK II, whereas Ser845 is phosphorylated by PKA (Jensen et al., 2003; Hu et al., 2007; O’Dell et al., 2010, 2015). GluR1 Ser831 phosphorylation potentiates single-channel conductance (Derkach, 2003), and GluR1 Ser845 phosphorylation increases the channel open probability (Banke et al., 2000).

Thus, changes in GluR1 Ser845 and Ser831 phosphorylation provide an indicator of synaptic plasticity. The aim of this study was to investigate whether NE influenced PTSD-like memory impairments *via* regulation of the β-AR-cAMP/PKA or CaMK II/PKC signaling pathway. We also observed the phosphorylation changes of Ser845 and Ser831 in GluR1.

## Materials and Methods

### Subjects

All male Sprague-Dawley rats (280–320 g) were purchased from the Experimental Animal Center at Sun Yat-sen University. Rats were individually on a 12-h light-dark cycle with *ad libitum* access to food and water. All behavioral experiments were performed during the light cycle between 9 AM and 3 PM. All procedures were approved by the Institutional Animal Care and Use Committee of the Zhongshan School of Medicine, Sun Yat-sen University, in accordance with the National Institutes of Health Guide for the Care and Use of Laboratory Animals.

### Surgery

The animals were adapted to the vivarium for at least 1 week before surgery. After each rat was fully anesthetized with sodium pentobarbital (50 mg/kg of body weight, i.p.), the skull was fixed to a stereotaxic frame (RWD, Shenzhen, China), and stainless-steel guide cannulas were implanted bilaterally with the cannula tips 2 mm above the BLA [coordinates: anteroposterior (AP), −2.8 mm from Bregma; mediolateral (ML), ±5.0 mm from midline; dorsoventral (DV), −6.5 mm from skull surface] according to the atlas of Paxinos and Watson (2007). The cannulas and two anchoring screws were affixed to the skull with dental cement. Stylets were inserted into the cannulas to maintain patency and were removed only for the infusion of drugs. The animals were allowed to recover for a minimum of 7 days before training and were handled for 2 min per day during this recovery period to accustom them to the infusion procedures.

### Fear Conditioning

After the handling days were completed, all rats were habituated in the acclimation chamber (Context A: an opaque PVC chamber, W × L × H: 30 cm × 24 cm × 21 cm, an opaque PVC floor, a brightness of 100 lux) for 4 min without shock exposure. The acclimation chamber was also cleaned with 4% acetic acid before each trial. Two days later, animals were trained in a fear conditioning chamber (Context B: a transparent Plexiglas chamber, W × L × H: 28 cm × 21 cm × 22 cm, a brightness of 60 lux) that contained a floor with 18 stainless steel rods and was connected to a shock generator and sound generator (Coulbourn Instruments, Allentown, PA, USA) developed in-house. The conditioning chamber was also cleaned with 70% ethanol before each trial. During training, each rat was placed into context B. After 110 s of free exploration, the rat was exposed to two footshocks (1.4 mA, 110 intertrial interval) that lasted for 3 s. After a 20 s delay, the rat received two tone cues (65 dB, 1 kHz, 30 s intertrial interval) that lasted for 15 s. After 20 s, the animals were placed back into its home cage. Twenty-four hours later, animals were tested in context A with cue trials (65 dB, 1 kHz, 2 kHz, or white noise) alone for 2 min. Two hours later, they were retested in context B for 2 min without the cues. Control groups were habituated to the training apparatus. The shock intensity (1.4 mA) was selected based on previous experiments conducted in our laboratory. In this case, animals will identify the conditioning context but not the cue as the right predictor of the shock. Freezing behavior was analyzed with a software program (Graphic State, Coulbourn Instruments, Allentown, PA, USA).

### Drug and Infusion Procedures

All drug solutions were freshly prepared on the experimental days. For the first experiment, the nonspecific β-AR antagonist DL-propranolol (0.5 μg/0.2 μl per hemisphere; Sigma Aldrich, St. Louis, MO, USA) either alone or together with NE (0.3 μg/0.2 μl per hemisphere; Sigma Aldrich, St. Louis, MO, USA) was dissolved in 0.9% saline and administered into the BLA immediately after conditioning with 1.4 mA. For the second experiment, different doses of NE (0.1, 0.3 or 1.0 μg/0.2 μl per hemisphere; Sigma Aldrich) were dissolved in 0.9% saline and administered into the BLA immediately after conditioning with 1.4 mA. These doses were selected on the basis of previous research (Quirarte et al., 1997; Banke et al., 2000; Barsegyan et al., 2014). For the last experiment, the selective PKA inhibitor Rp-cAMPS (4.0 μg/0.2 μl per hemisphere; Sigma Aldrich, St. Louis, MO, USA) or the selective CaMK II inhibitor KN-93 (5.0 μg/0.5 μl per hemisphere; Sigma Aldrich, St. Louis, MO, USA) was dissolved in saline and infused into the BLA 10 min before fear conditioning training. NE (0.3 μg/0.2 μl per hemisphere) was administered to the BLA immediately after conditioning with 1.4 mA. These doses were selected on the basis of previous research (Roozendaal et al., 2002; Arguello et al., 2014).

In each experiment, the injection needle protruded 2 mm beyond the tip of the cannulas, and a 0.2 μl or 0.5 μl injection volume was infused over a period of 1 min by an automated syringe pump CMA402 (CMA Microdialysis BA, Solna, Sweden). All drug solutions were prepared freshly before each experiment, and the infusion procedures used were identical to those described above. The injection cannulas were left in place for 1 min after drug infusion to maximize diffusion and to prevent backflow of the drug into the cannulas.

### Western Blot Analysis

After rats were sacrificed, brains were immediately frozen, and the BLA was microdissected using a 1 mm section rat brain matrix and frozen in liquid nitrogen prior to storage at −80°C. The supernatant was then assayed for total protein concentration using the BCA Protein Assay Kit. Tissue homogenate samples were resolved in 8% SDS-polyacrylamide gels, blotted electrophoretically onto PVDF membranes, blocked at room temperature for 1 h in PBS buffer containing 5% nonfat milk, and then blotted overnight at 4°C with antibodies to GluR1 (1:1,000; Millipore, Temecula, CA, USA), phosphorylated GluR1 at Ser831 and Ser845 (1:1,000; Millipore), and GAPDH (1/1,000; Cell Signaling Technology, Danvers, MA, USA). Then, the membranes were incubated with HRP-conjugated secondary antibody for 1 h. The densitometric analysis of Western blot was performed using a ChemiDoc XRS system (Bio-Rad, Hercules, CA, USA), and data analysis was performed by Image Lab version 5.2.1 (Bio-Rad, Hercules, CA, USA).

### Histology

After the testing sessions, each rat was deeply anesthetized with a moderate dose of sodium pentobarbital and transcardially perfused with 0.9% saline, followed by 4% paraformaldehyde. The animal was then decapitated, and its brain was removed from the skull and placed in 4% paraformaldehyde. After 7 days, the brains were sliced at 40 μm thickness and stained with thionin. The sites of microinjections were verified according to the atlas of Paxinos and Watson (2007). Rats with injection needle placements outside the BLA or with extensive tissue damage at the injection needle tips were excluded from analysis.

### Data Analysis

All data are expressed as the mean ± SEM. One-way ANOVA (*post hoc* Fisher’s least significant difference) and two-way ANOVA with Fisher’s PLSD *post hoc* tests were used when appropriate. Analyses were conducted using SPSS 20.0 software. *P* < 0.05 was chosen as the criterion for statistical significance.

## Results

### Histology

**Figure 1:**
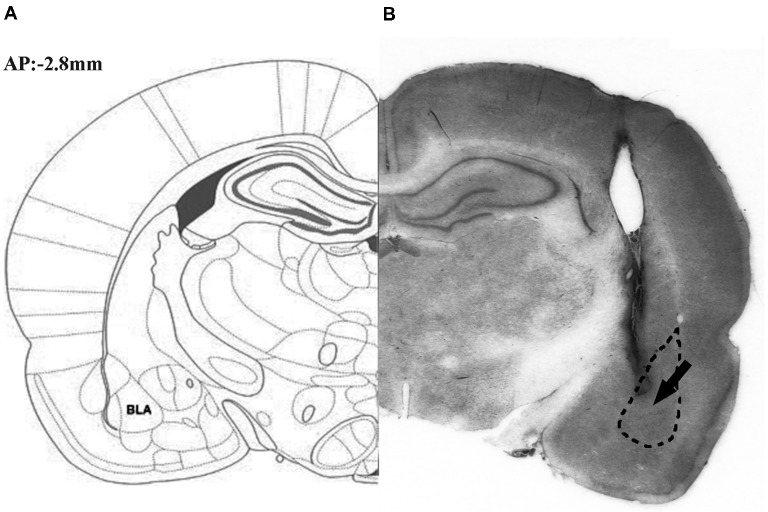
Representative photomicrograph illustrating the location of microinjection into the basolateral amygdale (BLA). The schematic (part **A**) and actual (part **B**) results on corresponding section taken from the rat brain atlas of Paxinos and Watson (2007), arrow points to needle tip.

Figure 1 illustrates the schematic (part **A**) and actual (part **B**) results of the corresponding sections taken from the rat brain atlas of Paxinos and Watson (2007).

### Intra-BLA Infusion of NE Induced Dose-Dependent PTSD-Like Memory Impairments

**Figure 2:**
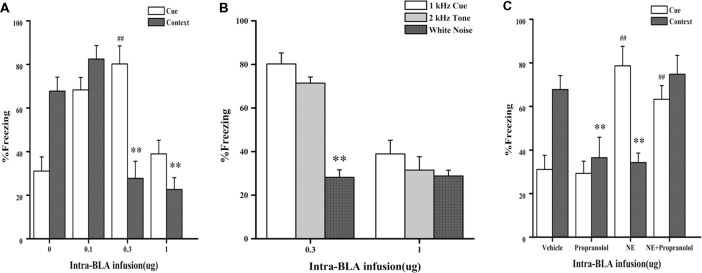
Intra-BLA infusion of norepinephrine (NE) induced a dose-dependent post-traumatic stress disorder (PTSD)-like memory impairments. **(A)** NE (0.3 μg/0.2 μl) administered into the BLA immediately after fear conditioning impaired retention of contextual fear memory and enhanced retention of cue fear memory. Concurrent infusion of β-adrenoceptor (β-AR) antagonist DL-propranolol (0.5 μg/0.2 μl) blocked this NE-induced memory impairments. Results represent mean ± SEM. **P < 0.01, ^##^*P* < 0.01 compared with vehicle. **(B)** NE (0.1, 0.3, or 1.0 μg/0.2 μl) administered into the BLA immediately after fear conditioning dose-dependent impaired retention of contextual fear memory. Results represent mean ± SEM. **P < 0.01, ^##^*P* < 0.01 compared with vehicle. **(C)** NE (0.3 μg/0.2 μl) induced PTSD-like memory impairments, which increased the response to 2 kHz tone but not to white noise. Results represent mean ± SEM. **P < 0.01 vs. 1 kHz cue.

In Experiment 1, we first tested whether NE-induced effects within the BLA had a similar dose-dependent effect as the GC-induced effects on PTSD-like memory impairment, we tested the effects of NE immediately after conditioning by microinfusing different doses (0.1, 0.3, and 1.0 μg) into the BLA of rats administered the high-intensity shock. As shown in Figure 2A, one-way ANOVA indicated that the 0.3 and 1.0 μg NE groups both showed impaired contextual fear conditioning, and the 0.1 μg NE group showed enhanced contextual and cue fear conditioning (context: *F*_(3,18)_ = 18.476, *P* < 0.001; cue: *F*_(3,18)_ = 14.929, *P* < 0.001). Fisher’s *post hoc* analysis confirmed that the 0.3 and 1.0 μg NE groups exhibited significantly decreased freezing time in the contextual memory retention test (0.3 μg NE: *P* < 0.001; 1.0 μg NE: *P* < 0.001). Fisher’s *post hoc* analysis also revealed that the 0.1 μg and 0.3 μg NE groups exhibited significantly increased freezing time in the contextual memory retention test (0.1 μg NE: *P* = 0.001; 0.3 μg NE: *P* < 0.001). We found that 0.3 and 1.0 μg NE can both impair contextual fear conditioning, and thus, we tested the generalization of fear responses to different cues. Only the 0.3 μg NE group of rats exhibited generalization of fear responses to different cues, as enhanced 1 kHz and 2 kHz cue fear conditioning was observed (*F*_(2,11)_ = 37.485, *P* < 0.001; Figure 2B).

Then, to investigate whether the effects of NE on memory was mediated by beta receptors or not, effective dose of NE with or without propranolol was microinfused into the BLA immediately after conditioning with a high-intensity shock (1.4 mA). As shown in Figure 2C, one-way ANOVA indicated that rats in the NE and propranolol groups both had impaired contextual fear conditioning; however, the NE + propranolol groups showed enhanced contextual and cue fear memories (context: *F*_(3,17)_ = 6.882, *P* = 0.003; cue: *F*_(3,17)_ = 13.459, *P* < 0.001). Fisher’s *post hoc* analysis confirmed that the NE and propranolol groups exhibited significantly decreased freezing time in the contextual memory retention test (NE: *P* = 0.008; propranolol: *P* = 0.008). Meanwhile, Fisher’s *post hoc* analysis also revealed that the propranolol and NE + propranolol groups exhibited significantly increased freezing time in the cue memory retention test (propranolol: *P* < 0.001; NE + propranolol: *P* = 0.006). Interestingly, when NE and propranolol were simultaneously injected into the BLA immediately after conditioning with the high-intensity shock (1.4 mA), freezing time increased in the contextual and cue memories retention test, indicating that this treatment enhanced contextual and cue fear memories.

Altogether, these results indicated that NE has a dose-dependent effect on PTSD-like memory impairments and that only the moderate dose (0.3 μg) of NE could induce PTSD-like memory impairments.

### NE Induced PTSD-Like Memory Impairments *via* Downregulation of AMPA Receptor Phosphorylation

**Figure 3:**
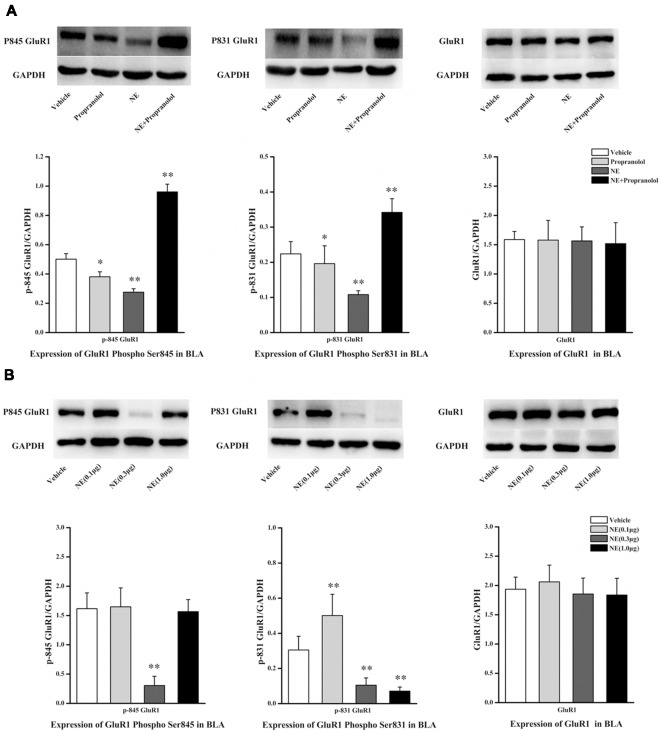
NE induced PTSD-like memory impairments *via* down-regulation the expression of Ser845 and Ser831 phosphorylation of GluR1. **(A)** Representative immunoblots showing the effect of the bilateral intra-BLA infusion of NE (0.3 μg/0.2 μl) or propranolol (5 μg/0.2 μl) immediately after fear conditioning induced change in phospho-GluR1 and total GluR1 levels. **(B)** Representative immunoblots showing the effect of the bilateral intra-BLA infusion of NE with the different doses of NE (0.1, 0.3, or 1.0 μg/0.2 μl) immediately after training induced change in phospho-GluR1 and total GluR1 levels. All results represent mean ± SEM. *P < 0.05, **P < 0.01, vs. vehicle group.

NE is mediated by mechanisms involving activation of β-ARs in the BLA, and the β-AR is directly linked to AMPAR functional modulation and trafficking to synaptic sites. GluR1 Ser831 and Ser845 phosphorylation sites have been proposed to play a key role in AMPAR trafficking and synaptic plasticity. GluR1 Ser831 is phosphorylated by PKC as well as other kinases, such as CaMK II, whereas Ser845 is phosphorylated by PKA. To further characterize the signaling events that lead to NE-induced PTSD-like memory impairments, we observed the phosphorylation changes of Ser845 and Ser831 in GluR1. As shown in Figure 3A, Ser831 phosphorylation of GluR1 was decreased in the NE and propranolol groups but was increased in the NE + propranolol group of rats (GluR1 Ser831: *F*_(3,12)_ = 21.291, *P* < 0.001; NE, *P* = 0.023; propranolol, *P* = 0.003; NE + propranolol, *P* = 0.004, LSD-t after one-way ANOVA). Ser845 phosphorylation of GluR1 was decreased in the NE and propranolol groups but was increased in the NE + propranolol group of rats (GluR1 Ser845: *F*_(3,12)_ = 28.524, *P* < 0.001; NE, *P* = 0.036; propranolol, *P* = 0.001; NE + propranolol, *P* = 0.001, LSD-t after one-way ANOVA). However, GluR1 was not altered in these three groups (*F*_(3,12)_ = 0.337, *P* = 0.779, one-way ANOVA).

Meanwhile, as shown in Figure 3B, Ser831 phosphorylation of GluR1 was decreased in the 0.3 μg and 1.0 μg NE groups but increased in the 0.1 μg NE group (*F*_(3,12)_ = 27.479, *P* < 0.001; 0.1 μg NE, *P* = 0.003; 0.3 μg NE, *P* = 0.003; 1.0 μg NE, *P* = 0.001, LSD-t after one-way ANOVA). Ser845 phosphorylation of GluR1 was decreased in the 0.3 μg NE group (*F*_(3,12)_ = 27.838, *P* < 0.001; 0.3 μg NE, *P* < 0.001, LSD-t after one-way ANOVA). GluR1 was not altered in these three groups (*F*_(3,12)_ = 0.59, *P* = 0.633, one-way ANOVA). Thus, phosphorylation of both Ser845 and Ser831 was decreased in the NE-induced PTSD-like memory impairment.

### NE Induced PTSD-Like Memory Impairments *via* Regulation of the cAMP/PKA and CaMK/PKC Signaling Pathways

**Figure 4:**
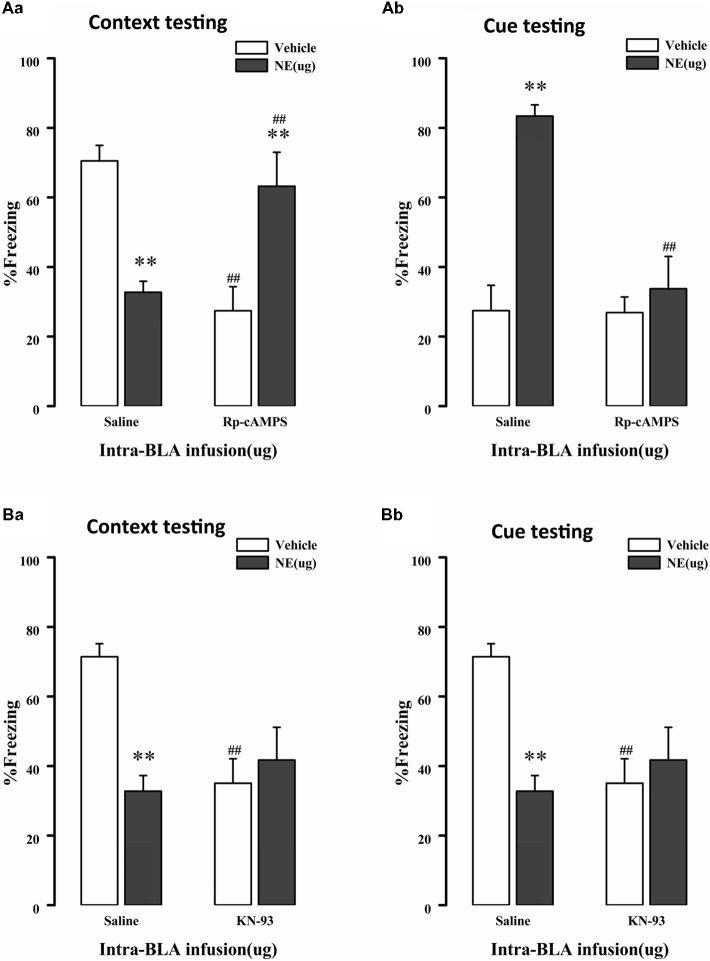
NE induced PTSD-like memory impairments *via* regulation 3′,5′-cyclic monophosphate (cAMP)/protein kinase A (PKA) and calcium/calmodulin-dependent protein kinase II (CaMK II)/PKC signal pathway. **(A)** The PKA inhibitor Rp-cAMPS (4.0 μg/0.2 μl) administered into the BLA 10 min before fear conditioning blocked the impairments of retention of contextual **(Aa)** and cue **(Ab)** fear memory induced by immediately post-training intra-BLA infusions of NE (0.3 μg/0.2 μl). **(B)** The CaMKII inhibitor KN-93 (5.0 μg/0.5 μl) administered into the BLA 10 min before fear conditioning also blocked the impairments of retention of contextual **(Ba)** and cue **(Bb)** fear memory induced by immediately post-training intra-BLA infusions of NE (0.3 μg/0.2 μl). All results represent mean ± SEM. **P < 0.01 compared with the corresponding vehicle group, ^##^*P* < 0.01 compared with the corresponding saline group.

As mentioned above, NE induces PTSD-like memory impairments along with decreased Ser845 and Ser831 phosphorylation of GluR1. Ser831 is phosphorylated by PKC as well as other kinases, such as CaMK II, whereas Ser845 is phosphorylated by PKA. Thus, to determine whether administration of NE within the BLA can activate the cAMP/PKA or CaMK II/PKC signaling pathway in PTSD-like memory impairments, we inhibited the cAMP/PKA or CaMK II/PKC pathway. As shown in Figures 4Aa,b, intra-BLA infusion of the PKA inhibitor Rp-cAMPS (4.0 μg) 10 min before fear conditioning blocked PTSD-like memory impairments induced by immediate post-training intra-BLA infusions of NE. The two-way ANOVA for percent freezing time during the contextual and cue memory retention test revealed a significant PKA inhibitor effect in the contextual memory retention test (context: *F*_(1,22)_ = 0.758, *P* = 0.395; cue: *F*_(1,22)_ = 12.541, *P* = 0.002), a significant effect of NE in the contextual and cue memory retention test (context: *F*_(1,22)_ = 0.020, *P* = 0.890; cue: *F*_1, 22_ = 19.605, *P* < 0.001), and a significant interaction between these two factors in the contextual and cue memory retention test (context: *F*_(1,22)_ = 25.607, *P* < 0.001; *F*_(1,22)_ = 11.988, *P* = 0.003). Rp-cAMPS not only induced contextual memory retention impairment when administered alone but also blocked PTSD-like memory impairments induced by immediate post-training intra-BLA infusion of NE (context: *F*_(3,19)_ = 8.533, *P* = 0.001; saline + NE vs. Rp-cAMPS + NE, *P* = 0.005, LSD-t after one-way ANOVA; cue: *F*_(3,19)_ = 15.15, *P* < 0.001; saline + NE vs. Rp-cAMPS + NE, *P* < 0.001, LSD-t after one-way ANOVA).

Meanwhile, as shown in Figures 4Ba,b, intra-BLA infusion of the CaMK II inhibitor KN-93 (0.5 μg) 10 min before fear conditioning blocked PTSD-like memory impairments induced by immediate post-training intra-BLA infusions of NE. The two-way ANOVA for percent freezing time during the contextual and cue memory retention test revealed a significant CaMK II inhibitor effect in contextual memory retention test (context: *F*_(1,24)_ = 3.036, *P* = 0.096; cue: KN-93: *F*_(1,24)_ = 21.788, *P* < 0.001), a significant effect of NE in the contextual and cue memory retention test (context: NE: *F*_(1,24)_ = 4.727, *P* = 0.041; cue: *F*_(1, 24)_ = 14.160, *P* = 0.001), and a significant interaction between these two factors in the contextual and cue memory retention test (context: *F*_(1, 24)_ = 8.535, *P* = 0.008; *F*_(1,24)_ = 19.372, *P* < 0.001). KN-93 not only induced contextual memory retention impairment when administered alone but also blocked PTSD-like memory impairments induced by immediate post-training intra-BLA infusion of NE (context: *F*_(3,21)_ = 4.708, *P* = 0.011; saline + NE vs. KN-93 + NE, *P* = 0.386, LSD-t after one-way ANOVA; cue: *F*_(3,21)_ = 18.786, *P* < 0.001; saline + NE vs. KN-93 + NE, *P* < 0.001, LSD-t after one-way ANOVA).

**Figure 5:**
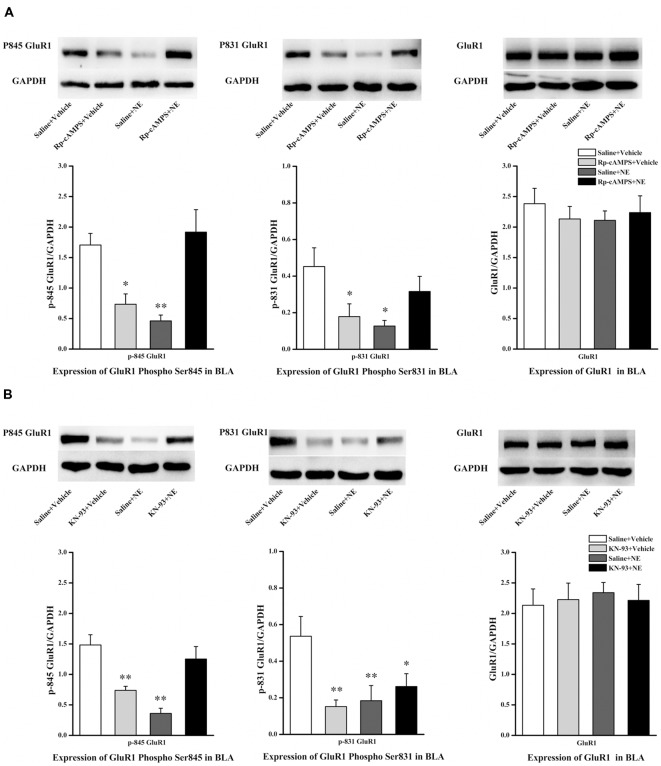
Inhibition cAMP/PKA or CaMK II/PKC signal pathway can down-regulate the expression of Ser845 and Ser831 phosphorylation of GluR1. Representative immunoblots showing the effect of the bilateral intra-BLA infusion of Rp-cAMPS (4.0 μg/0.2 μl; **A**) or KN-93 (5.0 μg/0.5 μl; **B**) 10 min before fear conditioning induced change in phospho-GluR1 and total GluR1 levels. All results represent mean ± SEM. *P < 0.05, **P < 0.01, vs. vehicle group.

We next tested the effect of inhibition of the cAMP/PKA or CaMK II/PKC signaling pathway in PTSD-like memory impairments on the phosphorylation changes of Ser845 and Ser831 of GluR1. We found that Ser831 phosphorylation of GluR1 was decreased in the saline + NE and Rp-cAMPS + vehicle groups (GluR1 Ser831: *F*_(3,12)_ = 3.69, *P* = 0.043; saline + NE, *P* = 0.011; Rp-cAMPS + vehicle, *P* = 0.026, LSD-t after one-way ANOVA; Figure 5A). Ser845 phosphorylation of GluR1 was decreased in the saline + NE and Rp-cAMPS + vehicle groups (GluR1 Ser845: *F*_(3,12)_ = 9.73, *P* = 0.002; saline + NE, *P* = 0.002; Rp-cAMPS + vehicle, *P* = 0.011, LSD-t after one-way ANOVA). However, GluR1 was not altered in these three groups (*F*_(3,12)_ = 0.302, *P* = 0.823, one-way ANOVA).

Additionally, Ser831 phosphorylation of GluR1 was decreased in the saline + NE, KN-93 + vehicle and KN-93 + NE groups compared with the saline + vehicle group (GluR1 Ser831: *F*_(3,12)_ = 4.879, *P* = 0.019; saline + NE, *P* = 0.008; KN-93 + vehicle, *P* = 0.005; KN-93 + NE, *P* = 0.030, LSD-t after one-way ANOVA; Figure 5B). Ser845 phosphorylation of GluR1 was decreased in the saline + NE and KN-93 + vehicle groups (GluR1 *F*_(3,12)_ = 12.465, *P* = 0.001; saline + NE, *P* < 0.001; KN-93 + vehicle, *P* = 0.003, LSD-t after one-way ANOVA). However, GluR1 was not altered in these three groups (*F*_(3,12)_ = 0.067, *P* = 0.977, one-way ANOVA).

Together, these results showed that intra-BLA infusion of KN-93 (0.5 μg) or Rp-cAMPS (4.0 μg) 10 min before fear conditioning can block PTSD-like memory impairments induced by immediate post-training intra-BLA infusions of NE, which can also lead to phosphorylation changes in the Ser845 and Ser831 sites of GluR1.

### Discussion

In PTSD patients, a core symptom is an excessive generalization of fear; patients show a strong response not only to a previous learned fearful cue but also cues that signal safety. This memory disturbances for the core traumatic event and peritraumatic cues, contributes to the intrusive recollection of traumatic event. In this study, we investigated whether NE was a key factor in such PTSD-like memory impairments, as well as the possible neurobiological mechanism. Researcher interest in this issue stems from recent reports showing that GCs could induce PTSD-like memory impairments (Kaouane et al., 2012). This animal model of PTSD-like memory impairment enables us to evaluate the ability of the individuals to restrict fear responses to the appropriate predictor of the threatening stimulus, instead of simply observe the freezing response to context or cue.

Evidence showed that GCs rely on NE activation in the BLA to affect memory consolidation (Banke et al., 2000; Roozendaal et al., 2006c). In this study, we found that only 0.3 μg NE microinfused into the BLA immediately after fear conditioning could induce PTSD-like memory impairments and simultaneously reduce GluR1 Ser845 and Ser831 phosphorylation. Furthermore, NE had a dose-dependent effect similar to that of GCs on PTSD-like memory impairments. We also found that intra-BLA infusion of a PKA inhibitor or CaMK II inhibitor before fear conditioning could block PTSD-like memory impairments induced by immediate post-training intra-BLA infusions of NE. Therefore, our findings suggested that the BLA noradrenergic system is involved in mediating PTSD-like memory impairments *via* regulation of the β-AR-cAMP/PKA and CaMK II/PKC signaling pathways.

Numerous studies using different experimental paradigms have shown that the amygdala is a key brain structure in modulation of the stress response and fear memory (Ehrlich et al., 2009; Hermans et al., 2014; Aubry et al., 2016). Emotionally arousing experiences are known to be associated with elevated levels of NE (Hatfield et al., 1999; McGaugh and Roozendaal, 2002), and BLA is a critical site of NE action (Roozendaal et al., 2008; Mueller and Cahill, 2010). We found that the β-AR antagonist propranolol or NE microinfused into the BLA immediately after fear conditioning could impair contextual and cue fear memory, which is consistent with previous evidence (Bush et al., 2010; Barsegyan et al., 2014; Zhou et al., 2015). Interestingly, when NE and propranolol were injected together into the BLA immediately after fear conditioning, contextual and cue fear memory was enhanced, one possible reason is that a low dose of propranolol only partly blocked the effect of NE-induced memory impairments. Because higher intensity of noradrenergic transmission required higher doses of the β-ARs antagonist than other tasks (Debiec and Ledoux, 2004). Another possibility is the involvement of α_2_-adrenoceptor. The α_2_ adrenoceptor is predominantly located on presynaptic noradrenergic terminals and its activation inhibits NE release (Langer, 1974; Talley et al., 1996). Ferry and McGaugh (2008) showed that post-training intra-BLA infusions of a selective α_2_ adrenoceptor agonist induced a dose-dependent impairment of retention of inhibitory avoidance. While the NE release onto β-AR is required for induction and maintenance of LTP (Roozendaal et al., 2006b; O’Dell et al., 2010), leading to retention enhancement. Further study using particularly β-ARs agonists is needed to clarify this issue.

Furthermore, we found that NE had a dose-dependent effect on PTSD-like memory impairments, and only a moderate dose of NE could induce PTSD-like memory impairments—low doses of NE enhanced contextual and cue fear memories, and high doses of NE impaired contextual fear memory. These findings are consistent with previous evidence showing that NE produced dose-dependent enhancement or impairment of memory in other experimental paradigms (Roozendaal et al., 2008; Li et al., 2011). NE or a β-ARs agonist infused into the BLA immediately post-training enhances the retention of emotionally arousing training experiences (Barsegyan et al., 2014), by regulating neural plasticity and information storage processes in other brain regions, including hippocampus (Atucha et al., 2017). However, direct effect of NE infusion into the hippocampus seems controversial. Atsak et al. (2012) indicated that post-training NE infusions into the dorsal hippocampus during retention of auditory fear conditioning has no significant effect on fear response. Our previous work (Zhu et al., 2018) suggested that intra-hippocampus infusion of propranolol, not NE, dose-dependently induced PTSD-like memory impairments. In addition, the PTSD-like memory impairment model used in this study can be regarded as the impairment of memory accuracy. Our results suggested that post-training NE infusions into the BLA decrease the accuracy of the fear memory, inconsistent with some others. This discrepancy may prominently due to our different experimental design. For example, Barsegyan et al. (2014) employed an object-in-context recognition memory design, had no emotional arousing in their study. Additionally, there are two factors in this fear conditioning, including context and cue, but only context in Atucha’ work (Atucha et al., 2017), and BLA is known for its crucial effect in cue fear memory. Moreover, the footshock in this work is 1.4 mA, much stronger than 0.6 mA they used, may induce more release of stress hormones, including GC, NE, etc. Moreover, another interesting finding is that the enhancement of irrelative cue retention performed earlier than impairment of context memory as the dose increases. It has long been known that BLA is crucially involved in the formation of cue fear memory (Ehrlich et al., 2009), thus changes may first show in cue memory. Since the memory specificity impaired after NE infusion, animals could not restrict fear responses to the appropriate predictor-context, but present higher freezing in cue test. NE regulates synaptic plasticity and glutamatergic excitatory post-synaptic currents (EPSCs; Almaguer-Melian et al., 2005; Walling et al., 2016) through several pathways that are regulated by phosphorylation of the AMPARs (Esteban et al., 2003; Oh et al., 2006). GluR1 Ser831 and Ser845 phosphorylation sites have been proposed to play a key role in AMPAR trafficking and synaptic plasticity. Moreover, prior findings indicated that NE signaling induces phosphorylation of the Ser845 and Ser831 sites of GluR1 in the emotional regulation of learning and memory (Banke et al., 2000; Derkach, 2003; Hu et al., 2007). However, how this regulation occurs in PTSD-like memory impairments is unknown. Our results showed that Ser831 and Ser845 phosphorylation of GluR1 was decreased by post-training intra-BLA infusions of 0.3 μg NE, and the phosphorylation decrease at the Ser831 or Ser845 site was driven by β-AR inhibition and infusion of high doses of NE, while phosphorylation increased at low doses, which was consistent with behavioral experiments indicating that there is an association between NE and AMPAR signaling pathways in the regulation of GluR1 Ser831 and Ser845 phosphorylation. This finding reveals that NE induces PTSD-like memory impairments *via* downregulation of AMPA receptor phosphorylation. In other words, this finding provides a potential explanation for the regulation of AMPA receptor trafficking and might offer a potentially beneficial treatment for PTSD.

It is well established that signaling molecules such as PKA, mitogen-activated protein kinase (MAPK), Ca^2+^/CaMKII have also been implicated in the maintenance of LTP and consolidation of fear memory in the amygdala (Toyoda et al., 2011). Indeed, cAMP/PKA signaling plays a critical role in presynaptically expressed LTP (Castillo et al., 2002; Young and Thomas, 2014). Increased PKA activity leads to phosphorylation of GluR1 on Ser845, increasing the channel open probability (Banke et al., 2000). While the increased CaMKII activity leads to phosphorylation of Ser831, potentiating single-channel conductance (Derkach, 2003). Furthermore, we found that intra-BLA infusion of KN-93 or Rp-cAMPS 10 min before fear conditioning blocked PTSD-like memory impairments induced by immediate post-training intra-BLA infusions of NE. This finding provided direct evidence for NE-induced PTSD-like memory impairments *via* regulation of the cAMP/PKA and CaMK II/PKC signaling pathways. Simultaneously, we also found Ser831 and Ser845 phosphorylation of GluR1 decreased due to pre-training intra-BLA infusions of the PKA inhibitor or CaMK II inhibitor alone. However, Ser831 and Ser845 phosphorylation of GluR1 was not significantly changed by pre-training intra-BLA infusions of Rp-cAMPS and post-training infusions of NE. These findings indicated that NE activation of β-ARs enhances behavioral memory *via* the cAMP/PKA and CaMK II/PKC signaling pathways (Hu et al., 2007; Zhou et al., 2013). However, besides activates PKA and CaMK II, other molecules including NMDA receptor, has been suggested to be the downstream targets of NE (Huang et al., 1993; Raman et al., 1996), which preferentially couple to phosphatases at lower levels of activation while activating kinases at higher levels. Vanhoose and Winder (2003) reported that a saturating dose of NMDA induces dephosphorylation of Ser845. Therefore, the changes in phosphorylation levels found in this study may be caused by a combination of factors. In future studies, it will be necessary to determine whether NMDAR involved in the modulation of NE induced PTSD-like memory impairment.

In conclusion, our findings reveal that NE regulates the β-AR-cAMP/PKA and CaMK II/PKC signaling pathways, leading to PTSD-like memory impairments.

## Author Contributions

HZ and LX contributed to the conception of the work. X-HL and R-TZ designed and collected the data. X-HL, R-TZ, BH, Y-WS and X-GW collected and analyzed the data. XL wrote the article. All authors discussed the results and commented on the manuscript. All authors approved the final version of the manuscript.

## Conflict of Interest Statement

The authors declare that the research was conducted in the absence of any commercial or financial relationships that could be construed as a potential conflict of interest.
